# Silencing long non-coding RNA CASC9 inhibits colorectal cancer cell proliferation by acting as a competing endogenous RNA of miR-576-5p to regulate AKT3

**DOI:** 10.1038/s41420-020-00352-5

**Published:** 2020-10-31

**Authors:** Hui-Zi Liu, Ti-Dong Shan, Yue Han, Xi-Shuang Liu

**Affiliations:** Department of Gastroenterology, The Affiliated Hospital of Qingdao University, Qingdao University, 16 Jiang Su Road, 262000 Qingdao, Shandong People’s Republic of China

**Keywords:** Colorectal cancer, Long non-coding RNAs

## Abstract

Increasing studies have shown that long non-coding RNAs (lncRNAs) are regarded as important regulators in the occurrence and development of colorectal cancer (CRC). Although lncRNA CASC9 has been studied in CRC, the detailed regulatory mechanism of CASC9 in CRC is still unclear. In this study, we found that CASC9 was significantly upregulated in CRC tissues and cell lines compared to normal controls and that aberrant expression was associated with the tumor-node-metastasis (TNM) stage of CRC. Functionally, CASC9 depletion efficiently inhibited the proliferation of CRC cells and induced cell apoptosis in vitro. Mechanistically, CASC9 was mainly enriched in the cytoplasm of CRC cells and interacted directly with miR-576-5p. Downregulation of miR-576-5p reversed the inhibitory effect of CASC9 siRNA on CRC cell progression. Furthermore, AKT3 has been identified as a downstream target of miR-576-5p. Spearman’s correlation analysis revealed that AKT3 was negatively correlated with miR-576-5p but positively correlated with CASC9. Downregulation of miR-576-5p restored the effect of CASC9 silencing on AKT3 expression. Therefore, silencing CASC9 could downregulate the expression of AKT3 by reducing the competitive binding of CASC9 to miR-576-5p, thus suppressing CRC cell proliferation and promoting cell apoptosis. In summary, we identified CASC9 as an oncogenic lncRNA in CRC and defined the CASC9/miR-576-5p/AKT3 axis, which might be considered a potential therapeutic target for CRC patients, as a novel molecular mechanism implicated in the proliferation and apoptosis of CRC.

## Introduction

Colorectal cancer (CRC) is one of the most frequent malignant tumors and remains the second leading cause of cancer-related mortality in the world^1,2^. Despite the fact that significant advances have been made in the diagnosis and treatment of CRC, the overall survival rate for patients has not improved distinctly. Therefore, it is indispensable to thoroughly understand the underlying pathogenesis of CRC development and identify effective therapeutic targets and prognostic biomarkers.

With the development of genomic and transcriptomic sequencing, it has been confirmed that only a small portion of the human genome is transcribed into protein-coding mRNAs, while most of the genome is transcribed into non-coding RNAs (ncRNAs)^3^. Long non-coding RNAs (lncRNAs) are a type of noncoding RNA with a length of more than 200 nucleotides^4^. Increasing evidence has indicated that lncRNAs participate in the pathophysiological processes of many diseases, including tumorigenesis and the malignant behavior of cancer^5^. MicroRNAs (miRNAs), another type of ncRNA with a length of 20–25 nucleotides, function in RNA silencing and posttranscriptional regulation of gene expression by binding to the 3′-untranslated regions (3′-UTRs)^6^. Numerous studies have confirmed that lncRNAs, as competing endogenous RNAs (ceRNAs), bind to miRNAs through complementary sequences, thereby attenuating the silencing effect of miRNA on target mRNA^7^.

Cancer susceptibility candidate 9 (CASC9), a confirmed lncRNA located on human chromosome 8q21.11, was initially identified as associated with esophageal squamous cell carcinoma^8^. Subsequent studies have shown that CASC9 promotes the progression of many other cancers, such as hepatocellular carcinoma, oral squamous cell carcinoma, CRC, and ovarian cancer^9–12^. Although it has been reported that CASC9, as an oncogene, can promote the growth of CRC in vivo and in vitro, the detailed mechanism remains unclear.

According to bioinformatic analysis, we focused on miR-576-5p, which has potential binding sequences with CASC9^13^. Previous studies have revealed that miR-576-5p plays an important role in CRC metastases. Compared with primary CRC tumors, miR-576-5p is significantly downregulated in the most common liver-metastatic carcinomas and is expressed at high levels in rare brain-metastatic carcinomas^14,15^. However, there is little research on the mechanism of miR-576-5p in CRC, which is worthy of further exploration.

The serine/threonine-protein kinase AKT, a critical protein in the PI3K/AKT signaling pathway, promotes cellular survival, proliferation, progression, and resistance to conventional chemotherapy in various kinds of human cancers^16^. Three AKT isoforms (AKT1, AKT2, and AKT3) have been identified in the mammalian genome. AKT1 is highly expressed and involved in the continuous proliferation of malignant tumors, including gastric cancer and lung cancer^17^. AKT2 has been reported to promote tumor development and progression in pancreatic cancer, ovarian cancer, and breast cancer^18^. AKT3 mainly acts by binding with miRNA, thus affecting tumor proliferation and apoptosis in thyroid cancer, oral squamous cell carcinoma, ovarian cancer, and CRC^19–22^.

In the present study, our data showed that CASC9 was highly expressed in CRC tissues and associated with the tumor-node-metastasis (TNM) stage. Furthermore, we found that the knockdown of CASC9 suppressed the proliferation and promoted the apoptosis of CRC cell lines. Finally, the mechanistic analysis revealed that CASC9 could act as a ceRNA, competitively binding with miR-576-5p and indirectly regulating the AKT3 gene.

## Results

### CASC9 is upregulated in CRC tissues and cell lines

**Fig. 1:**
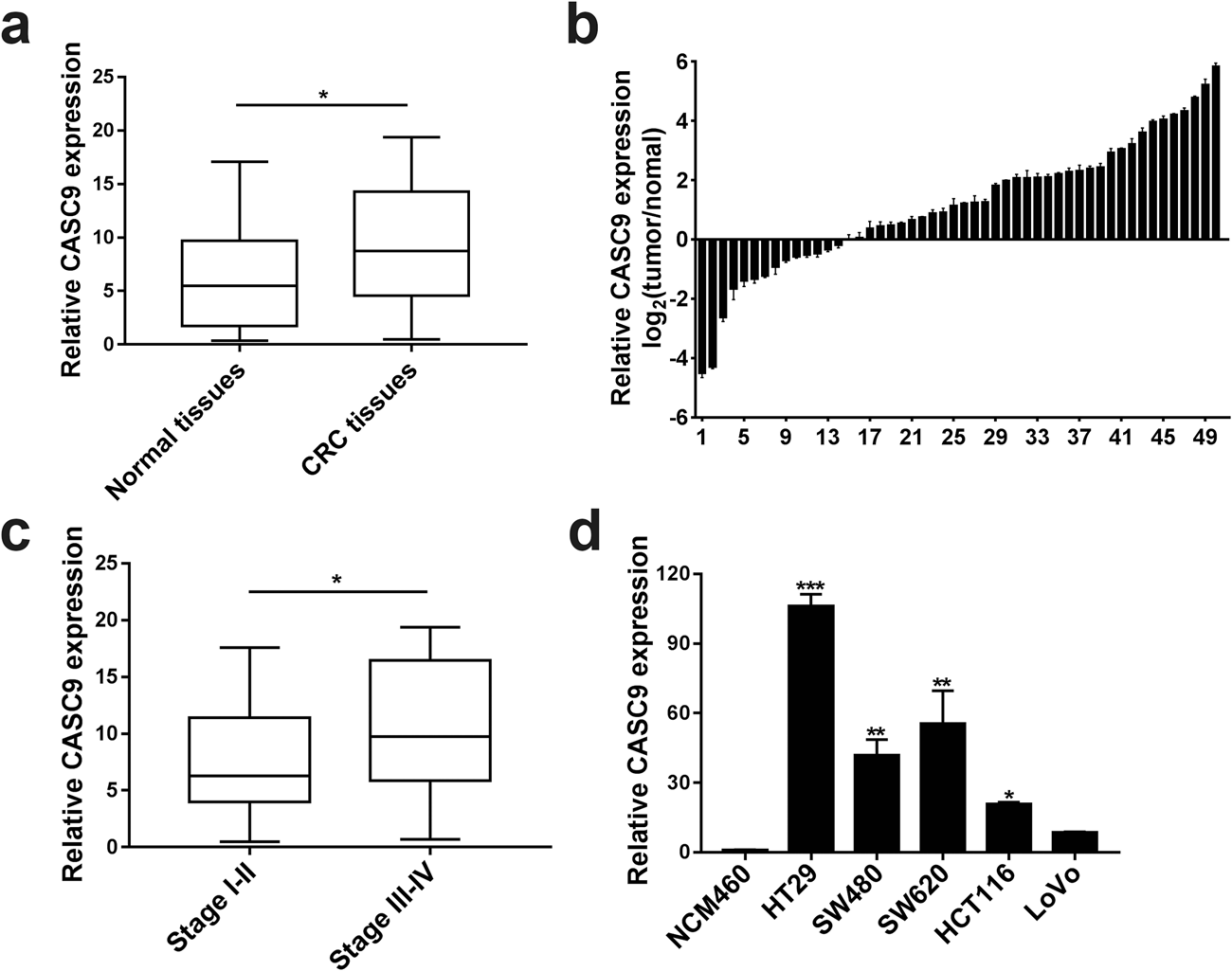
The differential expression of CASC9 in CRC tissues and cell lines. **a**, **b** The expression levels of CASC9 in 50 pairs of CRC and adjacent normal tissues were measured by RT-qPCR. **c** The expression levels of CASC9 in stage I–II and stage III–IV tumor tissues were detected. Statistical differences were analyzed using the Wilcoxon signed-rank test. **d** Relative expression of CASC9 in multiple CRC cell lines compared with NCM460 normal colonic epithelial cells was determined. Data are the mean ± SD of triplicate determinants (*P < 0.05, **P < 0.01, ***P < 0.001).

The expression levels of CASC9 were evaluated by RT-qPCR in 50 paired CRC samples and histologically normal adjacent tissues. The results showed that CASC9 was significantly overexpressed in tumor tissues (Fig. 1a, b). In addition, correlation analysis between CASC9 expression and the clinicopathological characteristics of CRC implied that CASC9 overexpression was related to the TNM stage (Table 1), and the CASC9 expression level was higher in stages III–IV than in stages I–II (Fig. 1c). In parallel, we examined the expression levels of CASC9 in different cell lines using RT-qPCR and found that compared with those in the normal colonic epithelial cell line NCM460, CASC9 expression levels were markedly increased in CRC cell lines such as HT-29, SW480, SW620, HCT-116, and LoVo (Fig. 1d). These results indicated that high CASC9 levels might play a nonnegligible role in the progression of CRC and may be useful as novel diagnostic and prognostic markers.

**Table 1:** Correlations between lncRNA CASC9 expression and clinicopathologic features in 50 colorectal cancer patients.

Clinicopathologic features	Total (*N* = 50)	CASC9 expression		*P* value (*χ*2 test)	Chi-square
		Low (*n* = 14, 28%)	High (*n* = 36, 72%)		
Age				0.684	0.166
<60	14	5	9		
≥60	36	9	27		
Gender				0.700	0.149
Male	30	9	21		
Female	20	5	15		
Tumor location				0.304	1.058
Colon	30	10	20		
Rectum	20	4	16		
Tumor size				0.574	0.315
<5 cm	29	9	20		
≥5 cm	21	5	16		
Depth of invasion				1.000	0.000
T1, T2	7	2	5		
T3, T4	43	12	31		
Lymphatic metastasis				0.095	2.794
Yes	20	3	17		
No	30	11	19		
Distant metastasis				0.676	0,174
Yes	7	1	6		
No	43	13	30		
TNM stage				0.03*	4.726
I–II	27	11	16		
III–IV	23	3	20		

*p < 0.05

### **Knockdown of CASC9** inhibits CRC cell proliferation via cell cycle arrest

**Fig. 2:**
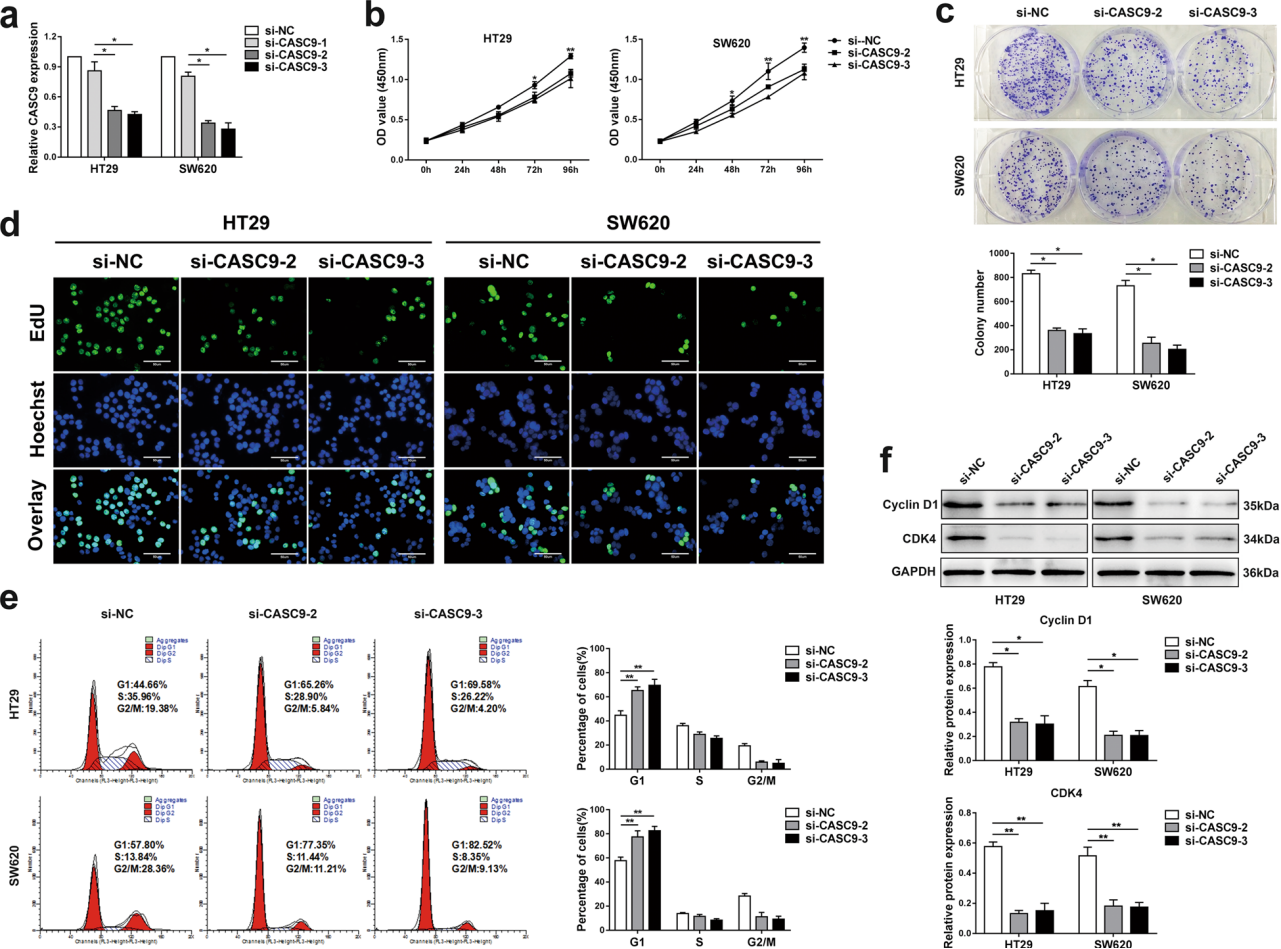
Knockdown of CASC9 inhibits CRC cell proliferation via cell cycle arrest. **a** CASC9 knockdown efficiencies were examined by RT-qPCR in HT29 and SW620 cells. **b** CCK-8 assays showed diverse growth curves of HT29 and SW620 cells after transfection with si-NC, si-CASC9-2, and si-CASC9-3. **c**, **d** The influence of CASC9 knockdown on CRC cell proliferation was indicated by colony formation and EdU assays. **e** Cell cycle of HT29 and SW620 cells transfected with si-NC, si-CASC9-2 and si-CASC9-3 was analyzed by flow cytometry. **f** The expression levels of cell cycle-related proteins (cyclin D1, CDK4) were detected by western blotting. Data are the mean ± SD of triplicate determinants (*P < 0.05, **P < 0.01).

To further investigate the role of CASC9 in the development of CRC cells, two CRC cell lines (HT29 and SW620) with high CASC9 expression levels were used as research objects. Next, HT29 and SW620 cells were transfected with three CASC9-targeting siRNAs (si-CASC9-1, si-CASC9-2, and si-CASC9-3) and a siRNA negative control (si-NC). RT-qPCR was used to examine the transfection efficiency, and the two siRNAs (si-CASC9-2 and si-CASC9-3) with higher transfection efficiency were used for further experiments (Fig. 2a). The CCK-8 assay was conducted to evaluate the proliferation capacity of CRC cells. The results showed that CASC9 knockdown inhibited cell viability (Fig. 2b). Similarly, colony formation and EdU assays also demonstrated that interference with CASC9 expression decreased cell proliferation compared to that in the control group (Fig. 2c, d).

To better understand the mechanisms of proliferation following CASC9 knockdown, flow cytometry was used to analyze the cell cycle distribution in siRNA-treated cell lines. Compared with cells treated with si-NC, both HT29 and SW620 cells treated with si-CASC9-2 and si-CASC9-3 showed distinctly increased cell percentages in G1 phase and decreased cell percentages in G2 phase (Fig. 2e). In addition, western blot analysis proved that the depletion of CASC9 reduced the expression levels of the cell cycle-related proteins cyclin D1 and CDK2, which are closely related to G1 cell cycle arrest (Fig. 2f). Therefore, these results suggested that knockdown of CASC9 might inhibit cell proliferation by regulating the cell cycle.

### Knockdown of CASC9 induces the apoptosis of CRC cells

**Fig. 3:**
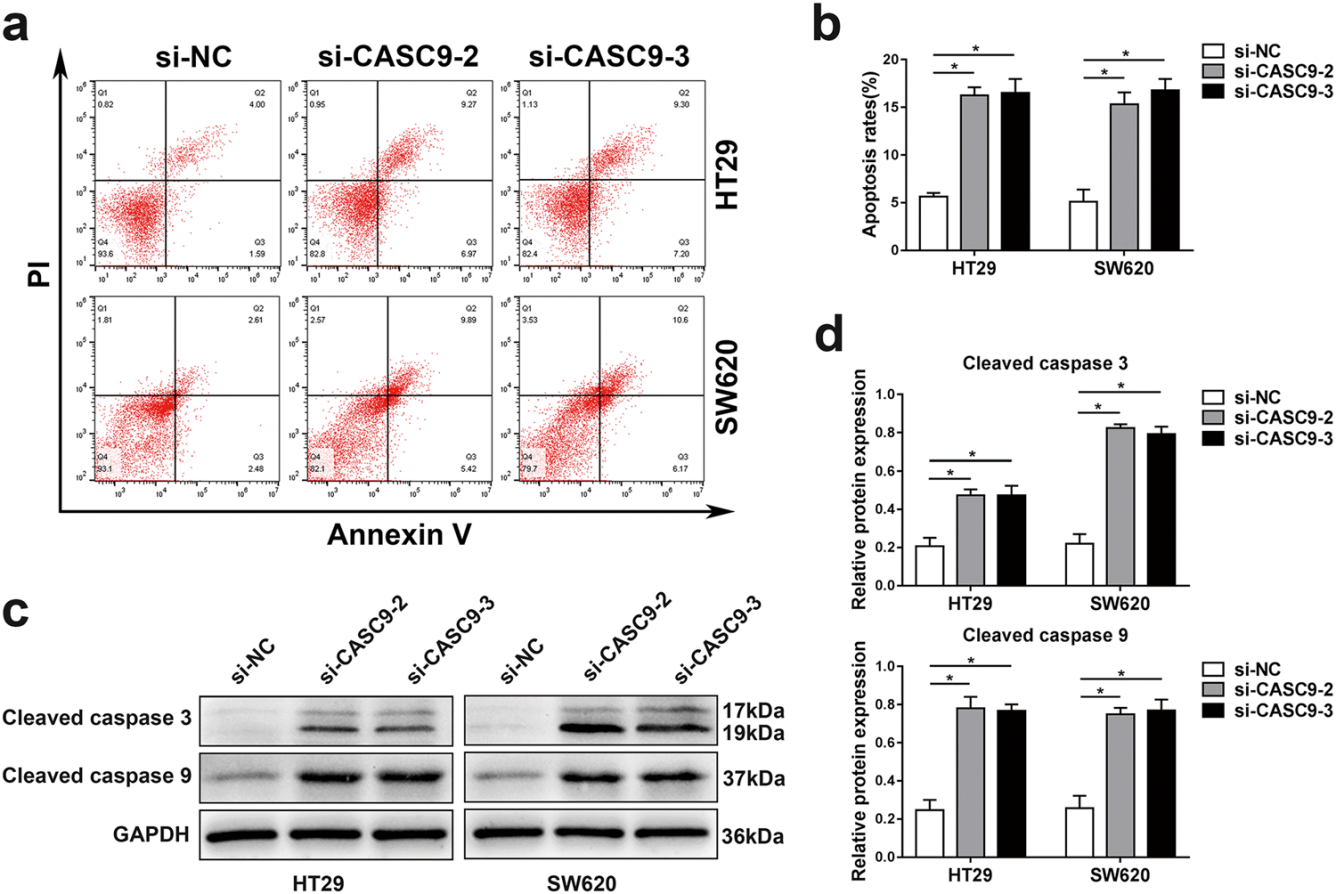
Knockdown of CASC9 promotes CRC cell apoptosis. **a**, **b** The apoptosis rates of HT29 and SW620 cells after siRNA treatment were detected by flow cytometry. **c**, **d** The expression levels of apoptosis-related proteins (cleaved caspase-3 and cleaved caspase-9) were determined by western blotting. Data are the mean ± SD of triplicate determinants (*P < 0.05).

To reveal the effect of CASC9 knockdown on CRC cell apoptosis, we measured the apoptosis level of CRC cells by flow cytometry. The results suggested that compared with the negative control group, the apoptosis rates of cell lines transfected with si-CASC9 were significantly increased (Fig. 3a, b). Furthermore, the alteration of apoptosis-related proteins was assessed using western blotting, and the levels of cleaved caspase-3 and cleaved caspase-9 were markedly elevated in the si-CASC9-transfected groups compared to the negative control group (Fig. 3c, d). Cleaved caspase-9 and caspase-3 are known to be prominent markers of the mitochondrion-mediated caspase-dependent pathway. Collectively, these data showed that knockdown of CASC9 could promote CRC cell apoptosis by activating the intrinsic apoptotic pathway.

### **CASC9 directly interacts with** miR-576-5p in CRC

**Fig. 4:**
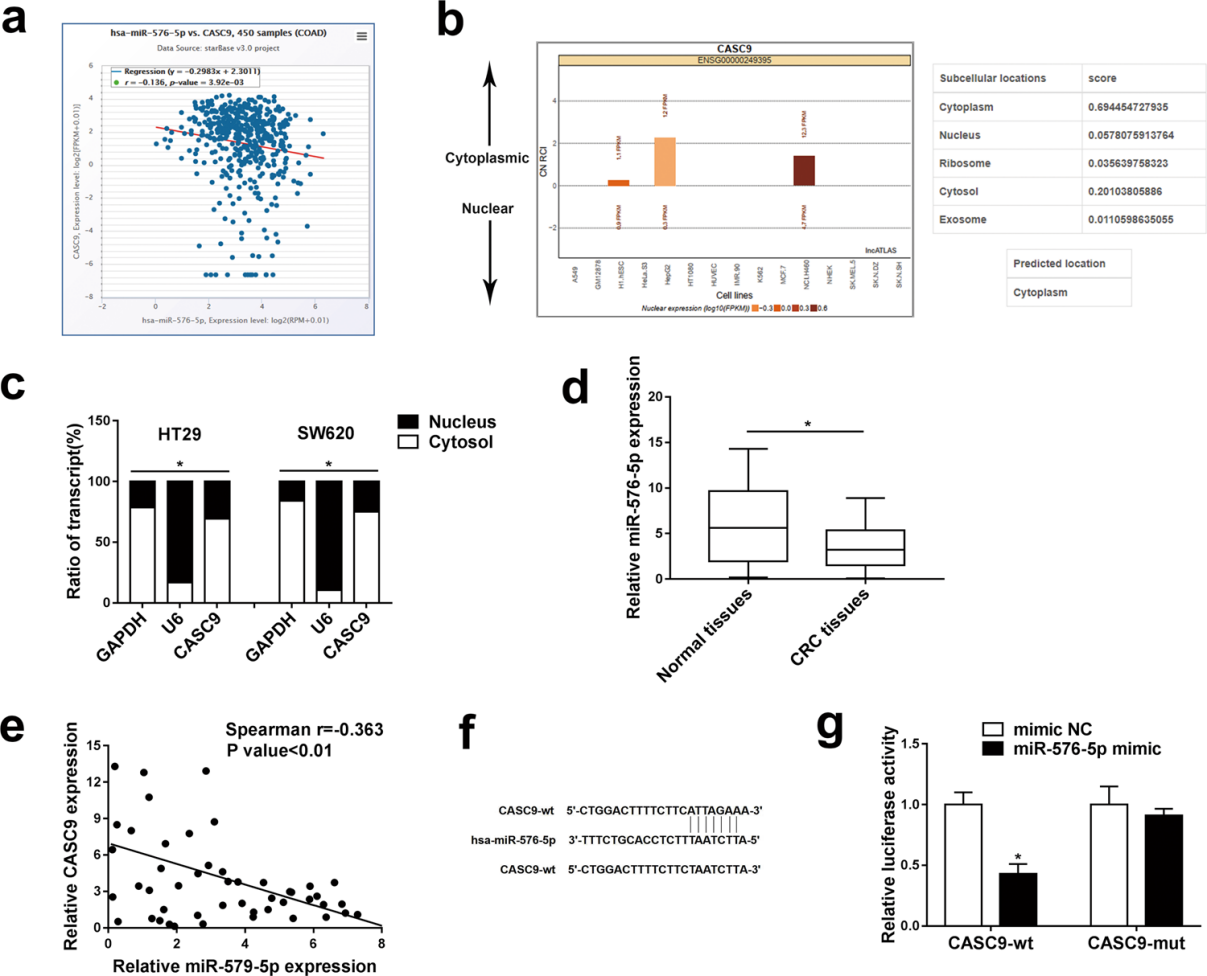
CASC9 is a direct target of miR-576-5p and is conversely related to miR-576-5p in CRC. **a** The correlation between CASC9 and miR-576-5p in CRC was predicted by bioinformatic analysis. **b** Subcellular localization of CASC9 was predicted by bioinformatics software. **c** Subcellular fractionation and RT-qPCR analysis confirmed that CASC9 was largely enriched in the cytoplasm of HT29 and SW620 cells. **d** The expression levels of miR-576-5p in CRC and adjacent normal tissues were measured by RT-qPCR. **e** Spearman’s correlation analysis showed a negative correlation between CASC9 and miR-576-5p in CRC tissues. **f** The predicted binding sites of miR-576-5p with the 3′-UTR of CASC9 are shown. **g** Luciferase reporter assay revealed the binding relationship between miR-576-5p and CASC9. Data are the mean ± SD of triplicate determinants (*P < 0.05).

Next, we aimed to clarify the regulatory mechanism of CASC9 in CRC development. miR-576-5p was predicted to be a potential target of CASC9 and negatively correlated with CASC9 using the online software program starBase (http://starbase.sysu.edu.cn) (Fig. 4a). Simultaneously, we found that CASC9 was mainly concentrated in the cytoplasm of most cells using the bioinformatics tool Lncatlas (http://lncatlas.crg.eu) (Fig. 4b). Subcellular fractionation was subsequently carried out to validate that CASC9 was principally located in the cytoplasm of HT29 and SW620 cells (Fig. 4c). Then, miR-576-5p expression levels were analyzed by RT-qPCR in 50 paired CRC samples and histologically normal adjacent tissues and the results showed that miR-576-5p was significantly reduced in tumor tissues (Fig. 4d). Furthermore, Spearman’s correlation analysis revealed a significant negative correlation between CASC9 and miR-576-5p in CRC tissues (Fig. 4e). To further analyze the relationship between CASC9 and miR-576-5p, wild-type lncRNA CASC9 (CASC9-wt) and mutant lncRNA CASC9 (CASC9-mut) luciferase reporter plasmids were constructed and cotransfected with miR-576-5p mimic into 293T cells (Fig. 4f). A luciferase reporter assay showed that overexpression of miR-576-5p distinctly suppressed the luciferase activity of the CASC9-wt group, whereas it had no effect on the luciferase activity of the CASC9-mut group (Fig. 4g). Taken together, these results revealed that miR-576-5p could inhibit CASC9 expression by directly targeting the 3′‐UTR of CASC9.

### **Suppression of miR-576-5p** reverses the inhibitory effect of CASC9 silencing on the growth of CRC cells

**Fig. 5:**
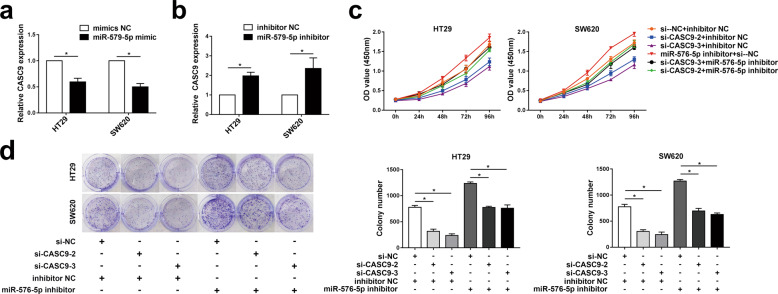
Suppression of miR-576-5p reverses the inhibitory effect of silencing CASC9 on the growth of CRC cells. **a**, **b** RT-qPCR was used to detect the expression levels of CASC9 in HT29 and SW620 cells after interference with miR-576-5p mimic or miR-576-5p inhibitor. **c**, **d** The proliferation of HT29 and SW620 cells transfected with si-CASC9 or miR-576-5p inhibitor was determined by CCK-8 and colony formation assay. Data are the mean ± SD of triplicate determinants (*P < 0.05).

We transfected HT29 and SW620 cells with miR-576-5p mimic or inhibitor to enhance or reduce miR-576-5p expression, respectively. RT-qPCR analysis suggested that the upregulation of miR-576-5p significantly decreased the expression level of CASC9 (Fig. 5a), while the downregulation of miR-576-5p increased the expression level of CASC9 (Fig. 5b). To verify whether CASC9 functions through miR-576-5p in CRC cells, we cotransfected si-CASC9 and miR-576-5p inhibitor into HT29 and SW620 cells, and the proliferation and apoptosis of these cells were assessed. CCK-8 assay showed that the cotransfection of si-CASC9 and miR-576-5p inhibitor promoted the proliferation of HT29 and SW620 cells in comparison to those in the si-CASC9 group (Fig. 5c). Similarly, colony formation assay also demonstrated that the miR-576-5p inhibitor promoted cell proliferation and reversed cell growth inhibition by si-CASC9 transfection in HT29 and SW620 cells (Fig. 5d). Overall, the miR-576-5p inhibitor reversed the inhibitory effect of si-CASC9 on CRC cell progression.

### **CASC9 regulates AKT3 expression via competitively binding with miR-576-5p** in CRC

**Fig. 6:**
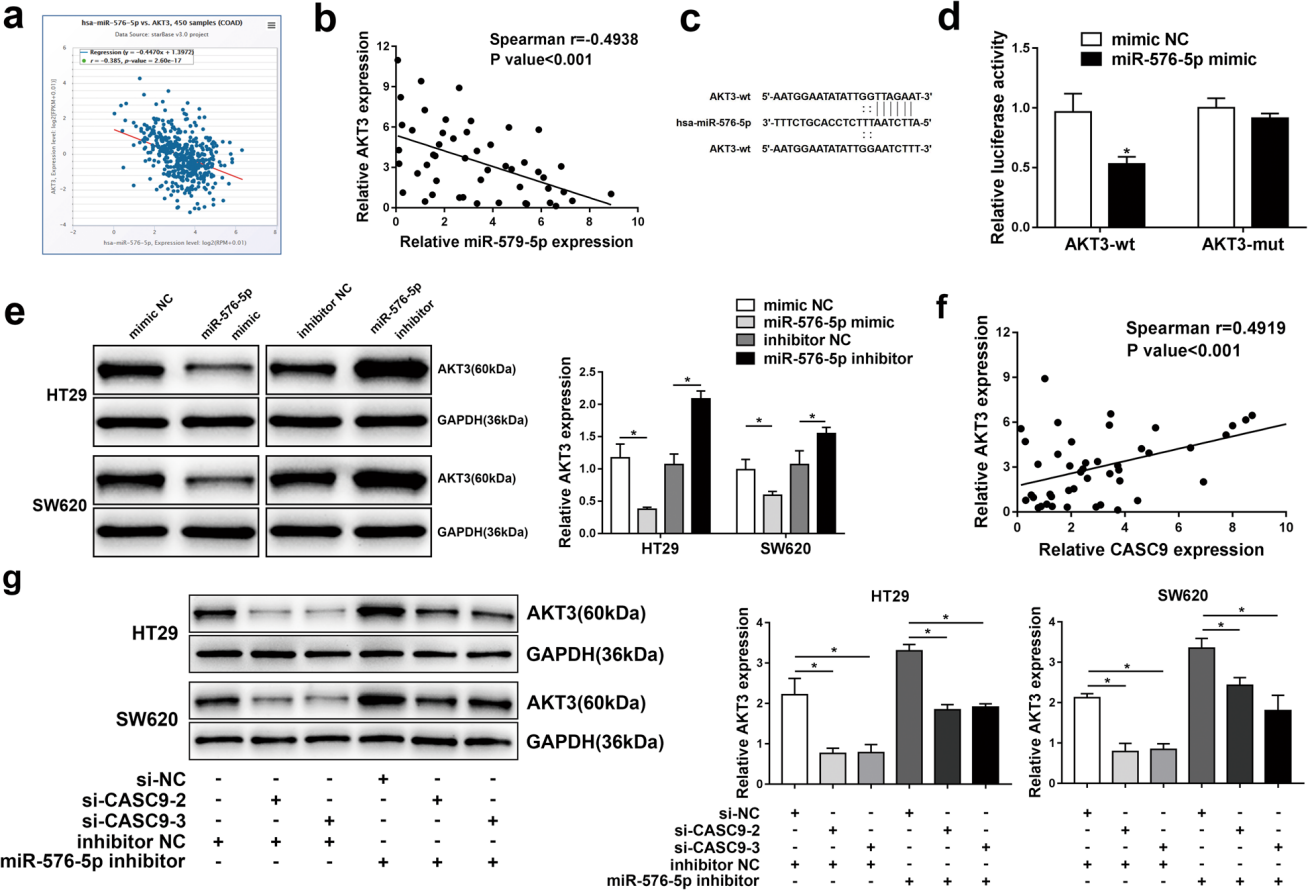
CASC9 regulates AKT3 expression via competitively binding with miR-576-5p in CRC. **a** The correlation between AKT3 and miR-576-5p in CRC was predicted by bioinformatic analysis. **b** The reverse association between AKT3 and miR-576-5p expression in CRC tissues was proven by Spearman’s correlation analysis. **c** The predicted binding sites of miR-576-5p with the 3′-UTR of AKT3 are shown. **d** Luciferase reporter assay revealed the binding relationship between miR-576-5p and AKT3. **e** western blot analysis showed the expression levels of AKT3 in HT29 and SW620 cells after transfection with miR-576-5p mimic or miR-576-5p inhibitor. **f** Spearman’s correlation analysis showed that AKT3 mRNA expression was negatively correlated with CASC9 in CRC tissues. **g** The expression levels of AKT3 in HT29 and SW620 cells transfected with miR si-CASC9 or miR-576-5p inhibitor were analyzed by western blotting. Data are the mean ± SD of triplicate determinants (*P < 0.05).

To further explore the molecular mechanism by which CASC9 affects the development of CRC, we used starBase (http://starbase.sysu.edu.cn), which predicted that AKT3 is a downstream target gene of miR-576-5p that is negatively related to miR-576-5p (Fig. 6a). The RT-qPCR results showed that AKT3 was inversely correlated with miR-576-5p via Spearman’s correlation analysis in CRC tissues (Fig. 6b). We constructed AKT3-wt and AKT3-mut luciferase reporter plasmids (Fig. 6c), and a luciferase reporter assay showed that miR-576-5p mimic transfection markedly restrained the luciferase activity of the AKT3-wt reporter in 293T cells (Fig. 6d). Afterwards, we transfected HT29 and SW620 cells with miR-576-5p mimic or inhibitor and determined the expression level of AKT3 by western blotting. The results suggested that overexpression of miR-576-5p reduced AKT3 expression, whereas inhibition of miR-576-5p improved the expression of AKT3 (Fig. 6e). To discover the relationship between CASC9 and AKT3, we used Spearman’s correlation analysis, which showed that the relative mRNA expression of AKT3 was positively correlated with that of CASC9 in CRC tissues (Fig. 6f). Western blot analysis revealed that knockdown of CASC9 apparently reduced AKT3 expression, which could be reversed by cotransfection with si-CASC9 and miR-576-5p inhibitor in HT29 and SW620 cells (Fig. 6g). These results demonstrated that CASC9 regulates AKT3 expression by sponging miR-576-5p in CRC.

## Discussion

As high-throughput sequencing technology has rapidly advanced, increasing numbers of lncRNAs have been recognized. Accumulating studies have demonstrated that aberrantly expressed lncRNAs are implicated in the occurrence and progression of CRC and could be considered biomarkers for diagnosis and prognosis as well as therapeutic targets^23^. For example, lncRNA CRNDE is upregulated in CRC compared to normal controls and facilitates the progression of CRC via miR-181a-5p-mediated activation of Wnt/β-catenin signaling^24^. SNHG1 exerts an oncogenic function in CRC and might act as a potential target for CRC diagnosis and treatment^25^. In recent years, a growing number of studies have shown that CASC9 is overexpressed in various cancers, contributing to the progression of cancer cells^26^. However, the role of CASC9 in CRC has rarely been reported. Therefore, we designed experiments to explore the effect of CASC9 on the proliferation and apoptosis of CRC and its potential mechanisms.

In this study, we found that the expression level of CASC9 was strikingly increased in CRC tissues compared with adjacent normal tissues and was positively related to the TNM stage, suggesting that CASC9 might be regarded as a diagnostic and prognostic marker. In vitro, we detected that compared to normal colonic epithelial cells, CASC9 expression was significantly upregulated in CRC cell lines. To further investigate the effect of CASC9 on the progression of CRC cells, we selected two cell lines with high CASC9 expression as research objects and conducted functional experiments after silencing CASC9 with siRNA. The results showed that CASC9 knockdown apparently suppressed the propagation of CRC cells by inducing cell cycle arrest at the G1 phase and promoting CRC cell apoptosis by activating caspase-9 and caspase-3, which indicated that CASC9 might be an important therapeutic target in CRC. Overall, these results demonstrate the carcinogenic effect of CASC9 on CRC.

CeRNA crosstalk between lncRNAs and mRNAs has been identified as an important regulatory mechanism in multiple diseases, including cancers^27^. The cytoplasmic localization of lncRNAs is conducive to their direct binding to miRNAs as ceRNAs or sponges, whereas nuclear localization might prevent ceRNAs from playing their role under steady-state conditions^28,29^. Recent studies have shown that CASC9 can regulate the progression of various human neoplasms by functioning as a ceRNA for miRNAs. LncRNA CASC9 facilitates glioma tumorigenesis by sponging miR-519d^30^. LncRNA CASC9 plays an oncogenic role in breast cancer by sponging the miR-195/497 cluster^31^. In this study, we first used bioinformatics tools to predict that CASC9 is predominantly localized to the cytoplasm of CRC cells, which was subsequently verified by a subcellular fractionation assay in CRC cells. Then, bioinformatics analysis showed that miR-576-5p might have putative binding sites with CASC9. We found that miR-576-5p was prominently downregulated and negatively correlated with CASC9 expression. Moreover, a luciferase reporter assay confirmed that CASC9 is a direct target of miR-576-5p. Furthermore, we realized that overexpression of miR-576-5p reduced CASC9 expression, whereas downregulation of miR-576-5p elevated CASC9 expression. After cotransfection of cells with miR-576-5p inhibitor and siRNA against CASC9, we analyzed the proliferation and apoptosis of CRC cells. The results revealed that inhibition of miR-576-5p could reverse the consequences of CASC9 silencing on CRC cell proliferation and apoptosis. Therefore, CASC9 knockdown can inhibit CRC cell proliferation and promote CRC cell apoptosis by reducing its targeted binding to miR-576-5p. To further explore whether CASC9 functions by affecting the distribution of miR-576-5p on its specific targets, we used bioinformatics tools to predict AKT3 as a potential target of miR-576-5p.

Recent studies indicated that AKT3 is distinctly overexpressed in CRC and that lncRNAs play an oncogenic role in CRC by functioning as a ceRNAs to modulate AKT3 expression^32,33^. In this research, a series of experiments proved that AKT3 is a target of miR-576-5p and is reciprocally associated with miR-576-5p. In addition, we found that AKT3 was positively related to CASC9 in CRC tissues. Finally, we observed that CASC9 knockdown inhibited AKT3 expression in vitro, but the alteration could be reversed by miR-576-5p inhibitor treatment. These results revealed that the effects of CASC9 on CRC proliferation and apoptosis could be partially responsible for sponging miR-576-5p and modulating AKT3 expression.

In conclusion, our study demonstrated that CASC9 is overexpressed in CRC tissues and cell lines compared to normal controls. In vitro, interference with CASC9 significantly inhibited the proliferation and promoted the apoptosis of CRC cells. Mechanistic experiments showed that CASC9 regulated CRC cell proliferation and apoptosis by acting as a ceRNA to sponge miR-576-5p and regulate its target AKT3, which has been proven to be an oncogene of CRC. Therefore, CASC9 might be considered a molecular marker for CRC treatment and prognosis.

## Materials and methods

### Samples from CRC patients

Fifty paired fresh CRC tumors and adjacent normal tissues were obtained from pathologically diagnosed patients who underwent surgical procedures at the Affiliated Hospital of Qingdao University. The collected surgical specimens were immediately frozen in a −80 °C freezer. On the basis of the International Union against Cancer (UICC), TNM staging (stage I, II, III, and IV) was performed on the samples. The study was conducted with the informed consent of all patients and the approval of the Ethics Committee of the Affiliated Hospital of Qingdao University.

### **Cell culture** and RNA interference

Human CRC cell lines (HT-29, SW480, SW620, HCT-116, and LoVo) and one normal colon epithelial cell line (NCM460) were purchased from the American Type Culture Collection (ATCC, USA) and cultured in DMEM or RPMI1640 (HyClone, Logan, UT, USA) medium with 10% fetal bovine serum (FBS, Gibco, Grand Island, NY, USA) in an incubator at 37 °C with 5% CO_2_.

Three small interfering RNAs (siRNAs) targeting lncRNA CASC9 (si-CASC9-1, si-CASC9-2, and si-CASC9-3), siRNA negative control (si-NC), miR-576-5p mimic, mimic negative control (mimic NC) miR-576-5p inhibitor, and inhibitor negative control (inhibitor NC) were synthesized and purified by GenePharma (Shanghai, China). The sequences of these RNAs are shown in Table 2. The oligonucleotides were transfected into the cells using Lipofectamine 3000 Reagent (Invitrogen, Carlsbad, CA, USA) according to the manufacturer’s protocol. The transfection efficiency was detected by RT-PCR.

**Table 2:** All primers and siRNA sequences used in this study.

Name	Sequence
For qRT-PCR	
GAPDH-F	GGGAGCCAAAAGGGTCATCA
GAPDH-R	TGATGGCATGGACTGTGGTC
U6-F	CTCGCTTCGGCAGCACA
U6-R	AACGCTTCACGAATTTGCGT
CASC9-F	TTGGTCAGCCACATTCATGGT
CASC9-R	AGTGCCAATGACTCTCCAGC
miR-576-5p	GGATTCTAATTTCTCCACGTCT
AKT3-F	TGTGGATTTACCTTATCCCCTCA
AKT3-R	GTTTGGCTTTGGTCGTTCTGT
siRNAs	
si-NC/mimic NC-F	UUCUCCGAACGUGUCACGUTT
si-NC/mimic NC-R	ACGUGACACGUUCGGAGAATT
si-CASC9-1-F	GGGCAUUGAGAAGUUAGAATT
si-CASC9-1-R	UUCUAACUUCUCAAUGCCCTT
si-CASC9-2-F	GGACUCAUAUUACCAGUCUTT
si-CASC9-2-R	AGACUGGUAAUAUGAGUCCTT
si-CASC9-3-F	GCUGCUUCCAUUCUAAACATT
si-CASC9-3-R	UGUUUAGAAUGGAAGCAGCTT
has-miR-576-5p mimic-F	AUUCUAAUUUCUCCACGUCUUU
has-miR-576-5p mimic-R	AGACGUGGAGAAAUUAGAAUUU
miRNA inhibitor NC	CAGUACUUUUGUGUAGUACAA
has-miR-576-5p inhibitor	AAAGACGUGGAGAAAUUAGAAU

### **Total RNA extraction and real-time quantitative PCR (RT-qPCR**)

Total RNA from clinical tissue and cell lines was extracted using TRIzol reagent (Invitrogen, Carlsbad, CA, USA) in accordance with the instructions. cDNA was synthesized with the PrimeScript RT reagent Kit (TAKARA, Dalian, China) or Mir-X miRNA First-Strand Synthesis Kit (TAKARA, Dalian, China). RT-qPCR was implemented with TB Green Premix Ex Taq II (TAKARA, Dalian, China) on the Roche LightCycler 480 System. GAPDH and U6 were used as the endogenous control genes of CASC9 and miR-576-5p, respectively. The relative expression was calculated with the 2^-ΔΔCT^ method. All the primer sequences are listed in Table 2.

### Subcellular fractionation

Cytoplasmic and nuclear RNA was extracted using the Nuclear and Cytoplasmic Protein Extraction Kit (Beyotime, Shanghai, China) and TRIzol reagent (Invitrogen, Carlsbad, CA, USA) according to the manufacturer’s instructions. RT-qPCR was used to measure the expression ratio of CASC9 between the cytoplasmic and nuclear fractions. GAPDH and U6 act as cytoplasmic and nuclear controls, respectively.

### Cell counting kit-8 (CCK-8) assay

CCK-8 (Meilun Biotechnology, Dalian, China) was used to detect the proliferation of CRC cells. During the experiment, cells were seeded in a 96-well plate at a density of 2 × 10^3^ cells per well. After attachment, cells were transfected with siRNA and incubated in an incubator at 37 °C with 5% CO_2_. At 24, 48, 72, and 96 hours after transfection, medium containing 10% CCK-8 was placed into each well, and the cells were incubated at 37 °C for 30 min. Then, the absorbance was detected at a wavelength of 450 nm with a microplate reader (Tecan, Lyon, France).

### Colony formation assay

After transfection with siRNA for 24 h, the cells were routinely trypsinized, seeded at a density of 1 × 10^3^ cells/well and cultured for 10–14 days in a six-well plate. Then, they were fixed with 4% paraformaldehyde and stained with crystal violet for visualization and counting. Experiments were performed at least three times.

### 5-Ethynyl-20-deoxyuridine (EdU) assay

Cell proliferation was also detected by the EdU assay using a BeyoClick™ EdU Cell Proliferation Kit with Alexa Fluor 488 (Beyotime Biotechnology, Shanghai, China). Cells were seeded and incubated in 96-well plates at 1 × 10^4^ cells/well. Forty-eight hours after transfection, the EdU assay was implemented according to the manufacturer’s protocol. In brief, cells were incubated with 10 μM EdU for 2 h and then fixed and stained with Click reaction mixture and Hoechst 33342. Images were taken with a fluorescence microscope (40 × 10), and the proportion of cells that incorporated EdU was calculated. Experiments were repeated in triplicate.

### Flow cytometry assay

Flow cytometry was used to detect cell apoptosis and the cell cycle. Before the assay, cells were harvested using trypsin and washed with ice-cold PBS twice at 48 h after transfection. Apoptosis assays were performed using an annexin V-FITC apoptosis assay kit (Absin, Shanghai, China). The harvested cells were resuspended in 300 μl binding buffer and successively stained with annexin V/FITC (5 μl, 15 min) and propidium iodide (PI, 5 μl, 5 min) in the dark at room temperature. Then, 100 μl of binding buffer was added to each tube, and all samples were analyzed by Apogee Flow Cytometers (Apogee Flow Systems, Hemel Hempstead, UK).

Cell cycle assays were performed using the Cell Cycle and Apoptosis Analysis Kit (Beyotime Biotechnology, Shanghai, China). After fixation overnight at −20 °C with 70% ethanol, the harvested cells were washed with PBS, resuspended in PI staining solution, incubated in a 37 °C water bath for 30 min, and then detected by MoFlo XDP (Beckman Coulter, Miami, FL, USA).

### Luciferase reporter assay

Oligonucleotides, which included human lncRNA CASC9 and the AKT3 3′-UTR target sequence binding to miR-576-5p, were cloned into the pmirGLO plasmids (Promega, Madison, WI, USA) to form the CASC9-wild-type (pmirGLO-CASC9-wt) and AKT3-wild-type (pmirGLO-AKT3-wt) reporter vectors. The corresponding mutants, which were named pmirGLO-CASC9-mut and pmirGLO-AKT3-mut, were used as negative controls. The reporter plasmids were cotransfected with miR-576-5p mimic using Lipofectamine 3000. Forty-eight hours after transfection, luciferase activity was assessed using a dual-luciferase reporter assay system (Promega, Madison, WI, USA). Experiments were repeated three times.

### Protein extraction and western blotting

Cells were washed three times with cold PBS and lysed on ice with RIPA buffer (Thermo Fisher Scientific, Waltham, MA, USA) containing a protease and phosphatase inhibitor cocktail. The concentrations of protein were determined using a BCA protein assay kit (Solarbio, Beijing, China). Equal amounts of protein were separated by SDS-PAGE and transferred electrophoretically onto PVDF membranes (Millipore, Bedford, MA, USA). The membranes were blocked in 5% skimmed milk powder dissolved in TBST for 2 h at room temperature and subsequently incubated overnight at 4 °C with the appropriate primary antibodies: anti-AKT3 (1:1000, Cell Signaling Technology, Danvers, MA, USA), anti-cyclin D1 (1:1000, Bioss, Beijing, China), anti-CDK4 (1:1000, Bioss, Beijing, China), anti-cleaved caspase-9 (1:1000, Cell Signaling Technology), anti-cleaved caspase-3 (1:1000, Cell Signaling Technology) and anti-GAPDH (1:10,000, Absin, Shanghai, China). Then, the membranes were washed with TBST three times and incubated for 90 min with a horseradish peroxidase (HRP)-conjugated secondary antibody at room temperature. The blots were detected with an ECL chemiluminescence solution and autoradiography with X-ray film. GAPDH was used as an internal control.

### **Statistical analysi**s

The data are expressed as the mean ± SD from three independent experiments. Differences between the two groups were analyzed using Student’s *t*-test. The correlations between lncRNA CASC9 expression and clinicopathologic features were determined by the chi-square test. Wilcoxon signed-rank test was used to compare RNA expression in CRC samples and its paired normal adjacent tissues. *p* values were all two-sided and a *p* value < 0.05 was considered to be statistically significant. The statistical analyses were performed using SPSS 22.0 (IBM, Armonk, NY, USA) and GraphPad Prism 7 (GraphPad Software, Inc., USA). A *p* value < 0.05 was considered to indicate statistical significance.
