# Correction to: Silencing long non-coding RNA CASC9 inhibits colorectal cancer cell proliferation by acting as a competing endogenous RNA of miR-576-5p to regulate AKT3

**DOI:** 10.1038/s41420-021-00564-3

**Published:** 2021-07-20

**Authors:** Hui-Zi Liu, Ti-Dong Shan, Yue Han, Xi-Shuang Liu

**Affiliations:** Department of Gastroenterology, The Affiliated Hospital of Qingdao University, Qingdao University, 16 Jiang Su Road, 262000 Qingdao, Shandong People’s Republic of China

**Keywords:** Colorectal cancer, Long non-coding RNAs

Correction to: *Cell Death Discovery* 10.1038/s41420-020-00352-5, published online 31 October 2020

Since publication of this article, the authors noticed that an incorrect image was used in Figure 3a si-NC/HT29 panel. The corrected image is shown below. The authors apologise for any inconvenience caused by this error. The original article has been corrected.

**Figure:**
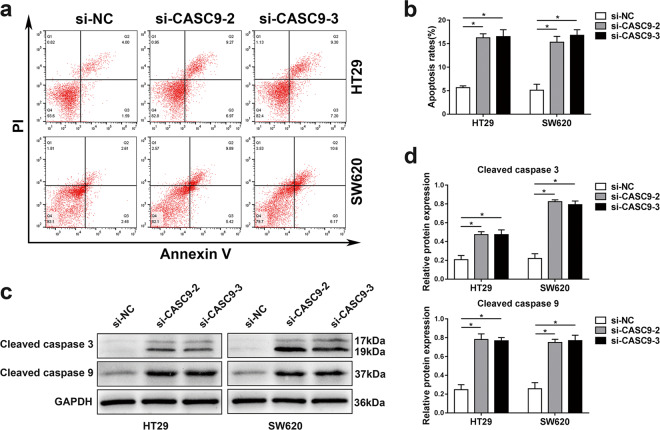
Fig. 3.

